# Respiratory dysfunction in a mouse model of spinocerebellar ataxia type 7

**DOI:** 10.1242/dmm.048893

**Published:** 2021-07-20

**Authors:** Anna F. Fusco, Logan A. Pucci, Pawel M. Switonski, Debolina D. Biswas, Angela L. McCall, Amanda F. Kahn, Justin S. Dhindsa, Laura M. Strickland, Albert R. La Spada, Mai K. ElMallah

**Affiliations:** 1 Department of Pediatrics, School of Medicine, Duke University, Durham, NC 27708, USA; 2 Department of Pathology & Laboratory Medicine, and Department of Neurology, School of Medicine, University of California Irvine, Irvine, CA 92697, USA; 3 Department of Neurology, School of Medicine, Duke University, Durham, NC 27708, USA; 4 Department of Medical Biotechnology, Institute of Bioorganic Chemistry, Polish Academy of Sciences, Noskowskiego 12/14 Str., 61-704 Poznan, Poland; 5 UCI Institute for Neurotherapeutics, Department of Neurology, School of Medicine, University of California Irvine, Irvine, CA 92697, USA

**Keywords:** SCA7, Spinocerebellar ataxia type 7, Respiratory, Phrenic, Hypoglossal

## Abstract

Spinocerebellar ataxia type 7 (SCA7) is an autosomal-dominant neurodegenerative disorder caused by a CAG repeat expansion in the coding region of the ataxin-7 gene. Infantile-onset SCA7 patients display extremely large repeat expansions (>200 CAGs) and exhibit progressive ataxia, dysarthria, dysphagia and retinal degeneration. Severe hypotonia, aspiration pneumonia and respiratory failure often contribute to death in affected infants. To better understand the features of respiratory and upper airway dysfunction in SCA7, we examined breathing and putative phrenic and hypoglossal neuropathology in a knock-in mouse model of early-onset SCA7 carrying an expanded allele with 266 CAG repeats. Whole-body plethysmography was used to measure awake spontaneously breathing SCA7-266Q knock-in mice at baseline in normoxia and during a hypercapnic/hypoxic respiratory challenge at 4 and 8 weeks, before and after the onset of disease. Postmortem studies included quantification of putative phrenic and hypoglossal motor neurons and microglia, and analysis of ataxin-7 aggregation at end stage. SCA7-266Q mice had profound breathing deficits during a respiratory challenge, exhibiting reduced respiratory output and a greater percentage of time in apnea. Histologically, putative phrenic and hypoglossal motor neurons of SCA7 mice exhibited a reduction in number accompanied by increased microglial activation, indicating neurodegeneration and neuroinflammation. Furthermore, intranuclear ataxin-7 accumulation was observed in cells neighboring putative phrenic and hypoglossal motor neurons in SCA7 mice. These findings reveal the importance of phrenic and hypoglossal motor neuron pathology associated with respiratory failure and upper airway dysfunction, which are observed in infantile-onset SCA7 patients and likely contribute to their early death.

## Abstract (precis)

**Summary:** A mouse model of spinocerebellar ataxia type 7 (SCA7) with at least 266 CAG repeats exhibits profound breathing deficits along with respiratory motor neuron degeneration and neuroinflammation.

## INTRODUCTION

Spinocerebellar ataxia type 7 (SCA7) is an autosomal-dominant neurodegenerative disorder characterized by progressive cerebellar dysfunction and loss of motor coordination (Benton et al., 1998; Lebre and Brice, 2003; Lindblad et al., 1996). SCA7 is also a polyglutamine disorder caused by a 37 to >400 CAG repeat expansion in the gene encoding ataxin-7 (*Atxn7*) (Benton et al., 1998; David et al., 1997; La Spada et al., 2001; Lebre and Brice, 2003; Seidel et al., 2012; Spada et al., 1991; Stoyas, 2018). The number of repeats positively correlates with symptom severity and inversely correlates with age of onset (Gouw, 1998; Johansson, 1998; Martin et al., 1999). Like other polyglutamine disorders, SCA7 exhibits anticipation, such that CAG repeat lengths may increase dramatically in successive generations and result in an accelerated onset of more severe disease (Gouw, 1998; van de Warrenburg et al., 2001). Consequently, parents may transmit a fatal infantile form of the disorder to their children before they begin presenting symptoms themselves (Ansorge et al., 2004; Gousse et al., 2018; Gouw, 1998; van de Warrenburg et al., 2001).

In addition to cerebellar dysfunction that results in ataxia, patients commonly develop cone-rod dystrophy, dysarthria, dysphagia and respiratory dysfunction (Benton et al., 1998; Johansson, 1998; La Spada et al., 2001; Michalik et al., 2004; van de Warrenburg et al., 2001). Children with infantile-onset SCA7 often succumb to aspiration pneumonia and respiratory failure, both of which are indicative of pathology in key respiratory centers (Ansorge et al., 2004; Gousse et al., 2018; Rüb et al., 2006, 2008; van de Warrenburg et al., 2001). SCA7 patients with dysphagia have brainstem degeneration in control centers important for swallowing, many of which are also critical for maintaining upper airway patency (Rüb et al., 2006). However, little is known about the features of upper airway dysfunction and respiratory failure in infantile-onset SCA7, and whether the respiratory motor neurons are affected.

To broaden our understanding of the impact of infantile-onset SCA7 on breathing, we evaluated respiratory output in awake spontaneously breathing SCA7-266Q knock-in mice at 4 and 8 weeks of age, before and after the onset of disease. In addition, we investigated motor neuron histology in putative phrenic and hypoglossal motor nuclei. The phrenic motor nuclei innervate the diaphragm, the primary muscle of inspiration. Neurodegeneration in phrenic motor neurons reduces respiratory capacity and leads to respiratory failure (Nicaise et al., 2012). The hypoglossal motor unit is essential for upper airway patency as it regulates the shape, stiffness and position of the tongue (Bailey, 2011). Contraction of the extrinsic tongue muscles preserves upper airway patency (Fuller et al., 1999). Hence, pathology in hypoglossal motor neurons results in dysphagia, dysarthria and aspiration pneumonia, all of which are prominent in infantile-onset SCA7 (Ansorge et al., 2004; Miller, 2008; van de Warrenburg et al., 2001). Although patients with infantile-onset SCA7 frequently die of respiratory failure, to date no group has investigated respiratory dysfunction or pathology in the phrenic or hypoglossal motor unit of a SCA7 animal model. Here, we describe the respiratory phenotype of the SCA7-266Q knock-in mouse model and find that progressive respiratory dysfunction and significant phrenic and hypoglossal motor neuron pathology are hallmarks of this disease.

## RESULTS

### SCA7-266Q mice experience progressive respiratory dysfunction

**Fig. 1.**
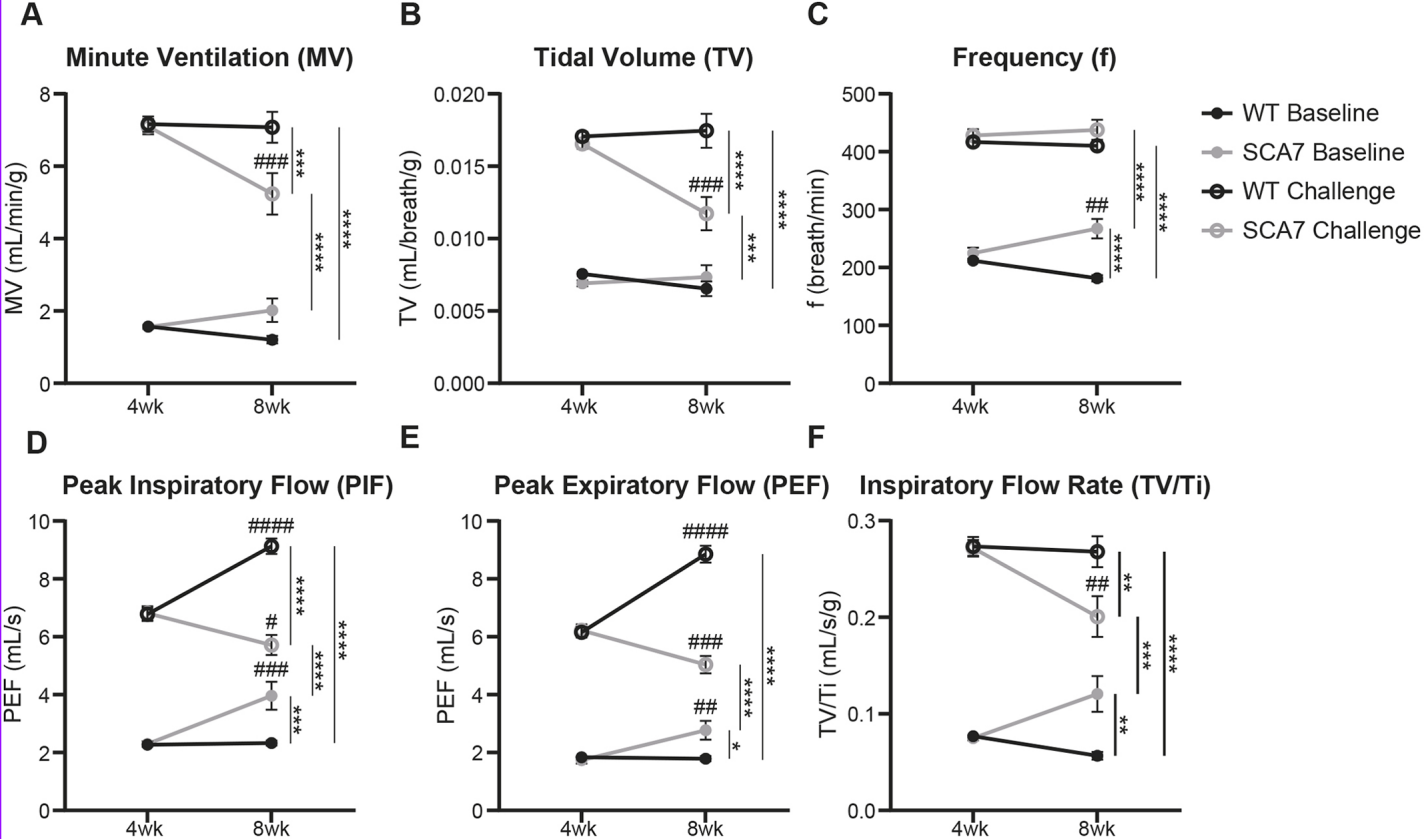
**SCA7 mice have pronounced respiratory deficits at 8 weeks of age.** (A-F) Measurements of respiratory function at baseline and during a hypercapnic/hypoxic challenge (FiCO_2_, 0.07; FiO_2_, 0.10; N_2_ balance) in wild-type (WT, black lines) and SCA7-266Q mice (gray lines) at 4 and 8 weeks of age. MV (A), TV (B), f (C), PIF (D), PEF (E) and TV/Ti (F) were comparable in wild-type and SCA7 mice at 4 weeks of age. However, at 8 weeks of age, SCA7 mice showed a compensatory increase in frequency (C), PIF (D), PEF (E) and TV/Ti (F) at baseline during normoxia. This increased work of breathing at rest foreshadows respiratory compromise appearing during the challenge. Compared to wild-type controls, the response of SCA7 mice to hypercapnia and hypoxia was significantly blunted, as shown by significant reductions in all measures except breath frequency. Wild-type respiratory parameters improve with age but SCA7 respiratory function was significantly reduced at 8 weeks compared to 4 weeks. Data are mean±s.e.m. Statistical significance (*/#) was determined using a two-way RM ANOVA, followed by a Bonferroni's post-hoc analysis (*/#*P*<0.05, **/##*P*<0.01, ***/###*P*<0.001, ****/####*P*<0.0001, *n*=10 mice per group). Asterisks (*) are shown for comparisons between groups at a single time point, and number signs (#) are shown for comparisons between time points for a single group.

Whole-body unrestrained plethysmography was used to assess respiratory function in unanesthetized SCA7 and wild-type mice at 4 and 8 weeks of age. At 4 weeks, no significant differences were observed between SCA7 and wild-type mice in any respiratory parameters at baseline or during a hypercapnic/hypoxic challenge (Fig. 1A-F; *P*>0.99 for all measures). When challenged, both wild-type and SCA7 mice significantly increased their respiratory output relative to baseline (Fig. 1A-F; *P*>0.99 for all measures). However, by 8 weeks, SCA7 mice began to show signs of significant respiratory compromise. At baseline, SCA7 mice displayed a compensatory increase in breath frequency (f, Fig. 1C; *P*=0.0042) and significant elevations in peak inspiratory flow (PIF, Fig. 1D; *P*=0.0001), peak expiratory flow (PEF, Fig. 1E; *P*=0.0021) and inspiratory flow rate (TV/Ti, Fig. 1F; *P*=0.0780) relative to their 4-week time point. In contrast, wild-type mice trended toward a decrease in breath frequency (Fig. 1C; *P*=0.0565) from 4 to 8 weeks of age but otherwise did not differ significantly in their respiratory parameters at baseline (Fig. 1A-F; *P*>0.99 for all measures). SCA7 mice showed respiratory impairment during a hypercapnic/hypoxic challenge at 8 weeks, as evidenced by significant reductions in MV (Fig. 1A; *P*=0.0005, *P*=0.0003), TV (Fig. 1B; *P*=0.0001, *P*<0.0001), PIF (Fig. 1D; *P*=0.0127, *P*<0.0001), PEF (Fig. 1E; *P*=0.0004, *P*<0.0001) and TV/Ti (Fig. 1F; *P*=0.0022, *P*=0.0017) relative to both their 4-week time point and age-matched wild-type mice (*P*-values included for each respective comparison). Wild-type controls by comparison showed significant increases in PIF (Fig. 1D; *P*<0.0001) and PEF (Fig. 1E; *P*<0.0001) but otherwise maintained comparable respiratory physiology from 4 to 8 weeks during a challenge (*P*>0.99 for all measures).

**Fig. 2.**
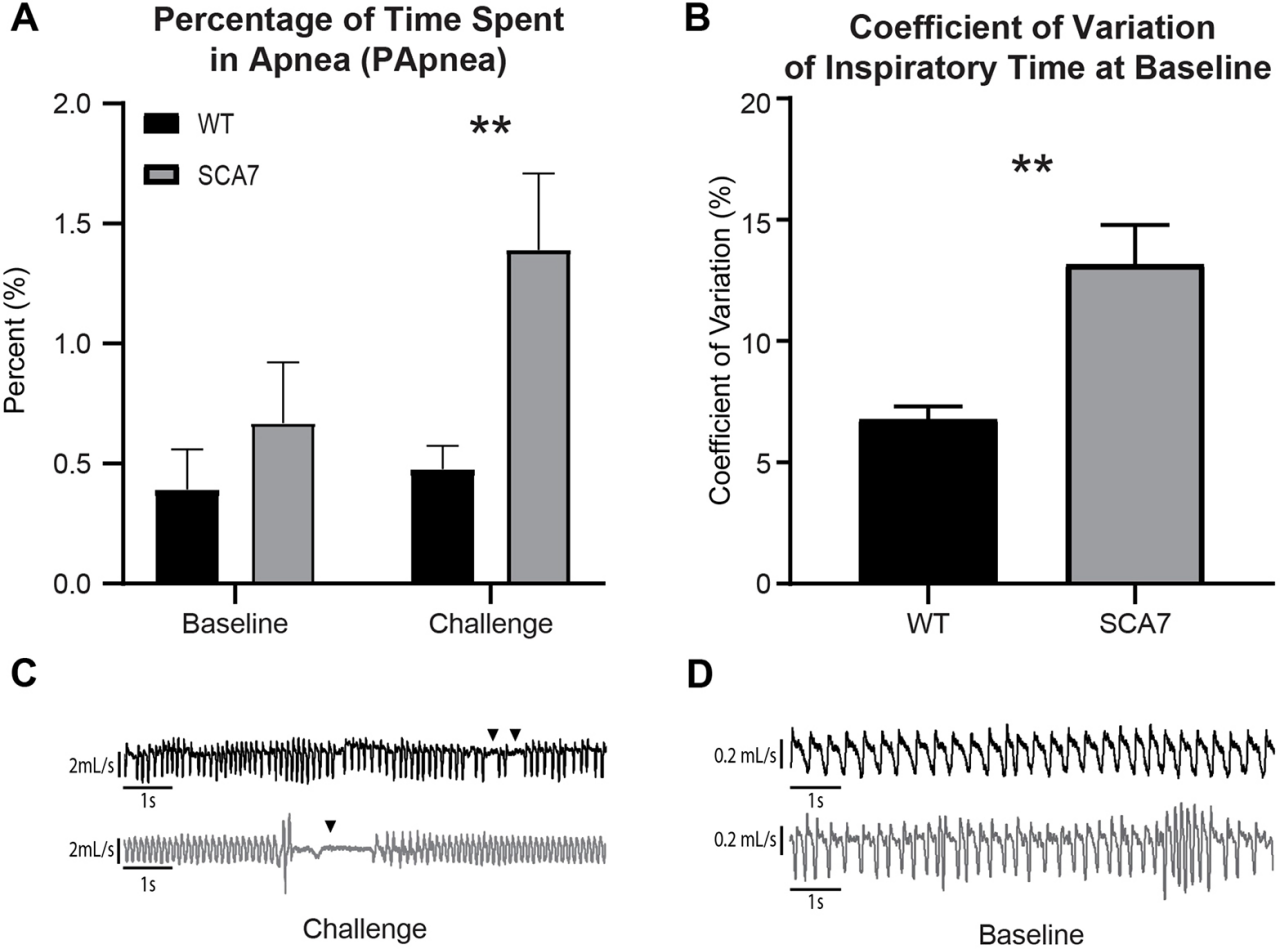
**SCA7 mice spend a greater percentage of time in apnea during a hypercapnic/hypoxic challenge, and breathe more erratically at baseline at 8 weeks of age.** (A) During the respiratory challenge at 8 weeks, SCA7 mice spent a greater percentage of time in apnea than wild-type (WT) mice. (B) During baseline breathing, SCA7 mice had an increased coefficient of variation of inspiratory time compared to wild-type mice. (C) A representative tracing of wild-type and SCA7 breathing during a challenge, chosen to represent the apneic episodes in SCA7 mice (indicated by black triangles). (D) A representative tracing of wild-type and SCA7 breathing during baseline chosen to illustrate the variability in inspiratory times in SCA7 mice. Data are mean±s.e.m. Statistical significance was determined using an unpaired two-tailed Student's *t*-test (**P<0.01, *n*=10 mice per group).

At 4 weeks of age, SCA7 mice spent less time in apnea (TApnea, *P*=0.0315) but did not differ significantly from wild-type mice in other measurements of apnea during a respiratory challenge (results not shown). However, by 8 weeks, SCA7 mice spent a greater percentage of time in apnea (PApnea, Fig. 2A; *P*=0.01) and displayed a trend toward a greater quantity (NApnea, *P*=0.1957) and duration of apneas (TApnea, *P*=0.0770) when challenged (results not shown). In addition, SCA7 mice trended toward less erratic breathing at baseline than wild-type mice at 4 weeks (*P*=0.0721), but by 8 weeks, their breathing was more erratic than wild-type controls, as evidenced by a significantly higher coefficient of variation of inspiratory time (Fig. 2B; *P*=0.0014). In summary, 8-week-old SCA7 mice exhibited longer apneas during the challenge (Fig. 2C) and increased breathing irregularity at baseline (Fig. 2D) compared to wild-type mice. Altogether, these findings are indicative of progressive respiratory pathology, which appears in SCA7-266Q mice by 8 weeks of age.

### SCA7-266Q mice show motor neuron pathology in phrenic and hypoglossal nuclei

**Fig. 3.**
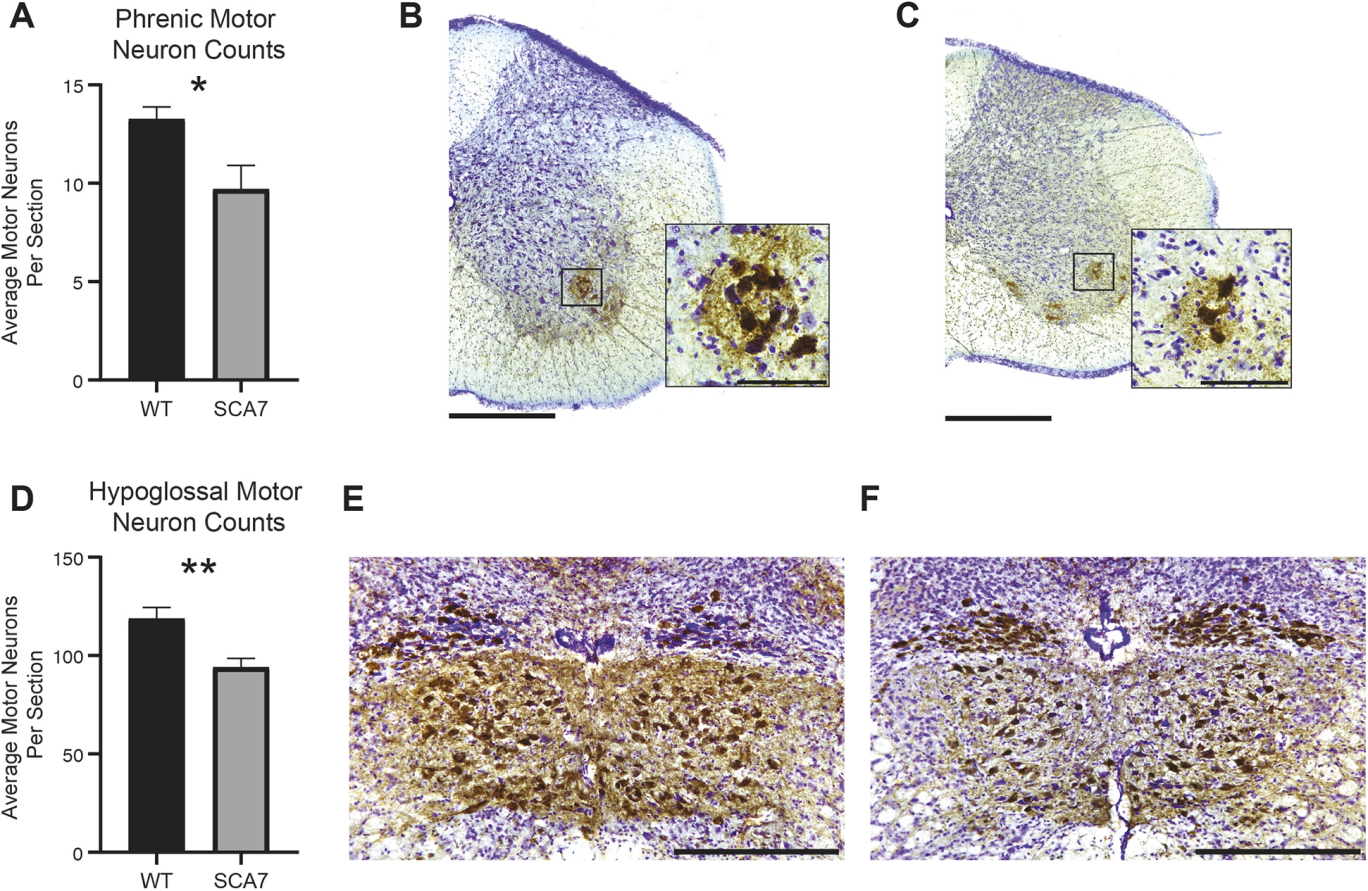
**SCA7 mice show motor neuron degeneration in the putative phrenic and hypoglossal nuclei.** Quantification of motor neurons (ChAT^+^ cells) from putative phrenic (A) and hypoglossal motor nuclei (D) of 10-week-old wild-type (WT) and SCA7 mice. (B,C) Representative images of ChAT antibody staining with Cresyl Violet of wild-type (B) and SCA7 (C) cervical spinal cord with a magnified region of the putative phrenic motor neuron. Representative images of ChAT antibody staining with Cresyl Violet of wild-type (E) and SCA7 (F) medulla for hypoglossal motor neurons. Data are mean±s.e.m. Statistical significance was determined using an unpaired two-tailed Student's *t*-test (*P<0.05; **P<0.01; phrenic, *n*=6 wild type, *n*=4 SCA7; hypoglossal, *n*=4 wild type, *n*=5 SCA7). Scale bars: 400 µm (gross) and 90 µm (magnified) (B,C); 360 µm (E,F).

**Fig. 4.**
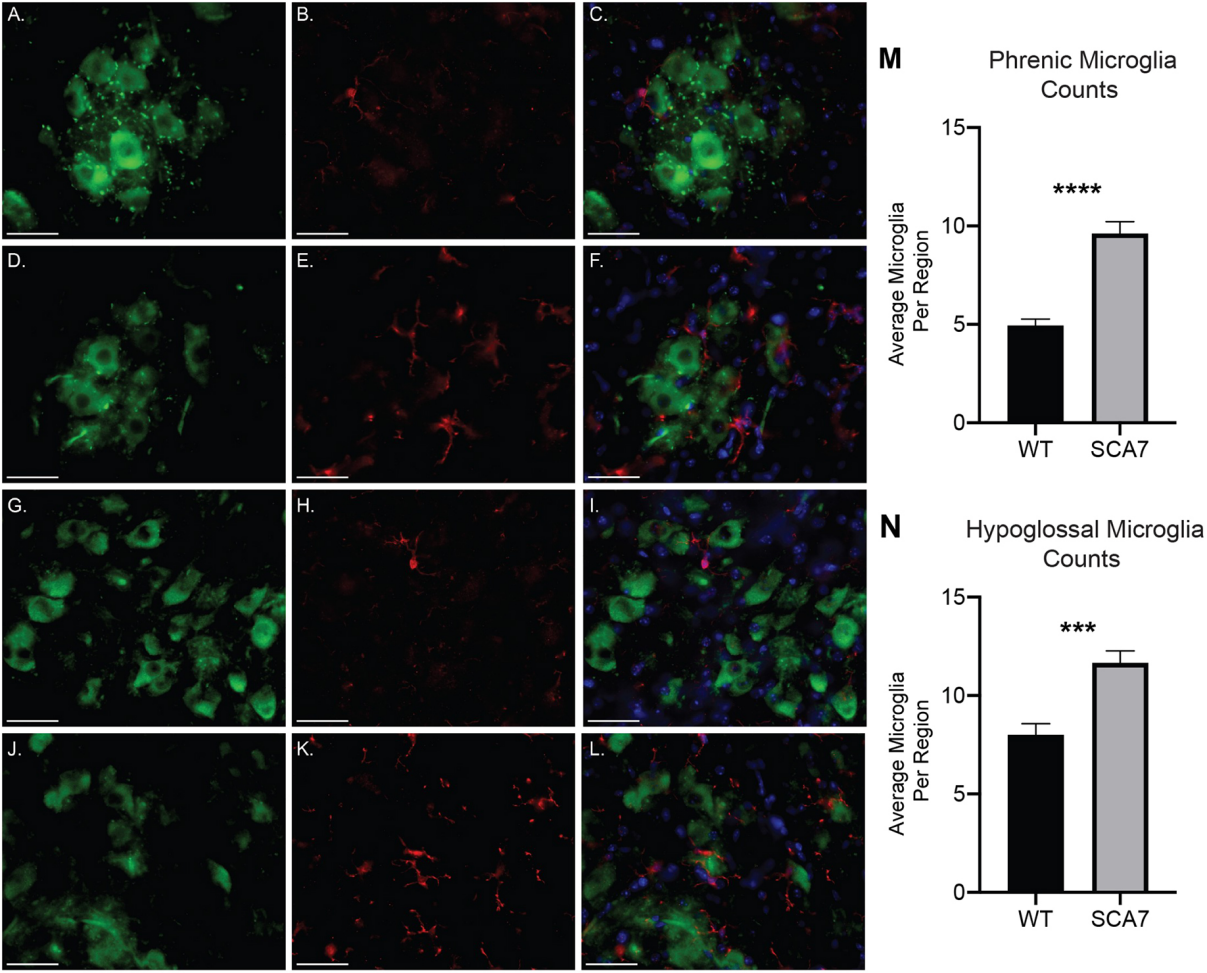
**SCA7 mice exhibit increased amounts of activated microglia in the putative phrenic and hypoglossal nuclei.** (A-L) Representative images of ChAT- (green), Iba1- (red) and DAPI- (blue) stained cervical spinal cord and medulla of 10-week-old wild-type (WT) and SCA7 mice. Putative phrenic motor nucleus of wild-type (A-C) and SCA7 (D-F) mice. Hypoglossal motor nucleus of wild-type (G-I) and SCA7 (J-L) mice. Quantification of Iba1^+^ cells revealed a significant increase in the number of microglia in putative phrenic (M) and hypoglossal (N) motor nuclei of SCA7 mice compared to wild-type mice. Data are mean±s.e.m. Statistical significance was determined using an unpaired two-tailed Student's *t*-test (***P<0.001, ****P<0.0001; phrenic, *n*=4 wild type, *n*=4 SCA7; hypoglossal, *n*=3 wild type, *n*=5 SCA7). Scale bars: 40 µm.

To determine whether respiratory insufficiency correlates with neurodegeneration and neuroinflammation of phrenic and hypoglossal motor neurons, we quantified putative motor neurons and microglia in the relevant motor nuclei at 10 weeks of age, near disease end stage. Compared to wild-type controls, SCA7 mice have significant reductions in both putative phrenic (Fig. 3A-C; *P*=0.02) and hypoglossal (Fig. 3D-F; *P*=0.01) motor neurons. To assess neuroinflammation, we performed dual staining with anti-ChAT, to label motor neurons, and anti-Iba1, to label microglia. In SCA7, Iba1^+^ cells with the morphology of activated microglia surrounded putative phrenic and hypoglossal motor neurons (Fig. 4A-L). When quantified, the number of microglia in the regions of the putative phrenic and hypoglossal motor nuclei was elevated in SCA7-266Q mice (Fig. 4M,N; *P*<0.0001 and *P*<0.001, respectively).

**Fig. 5.**
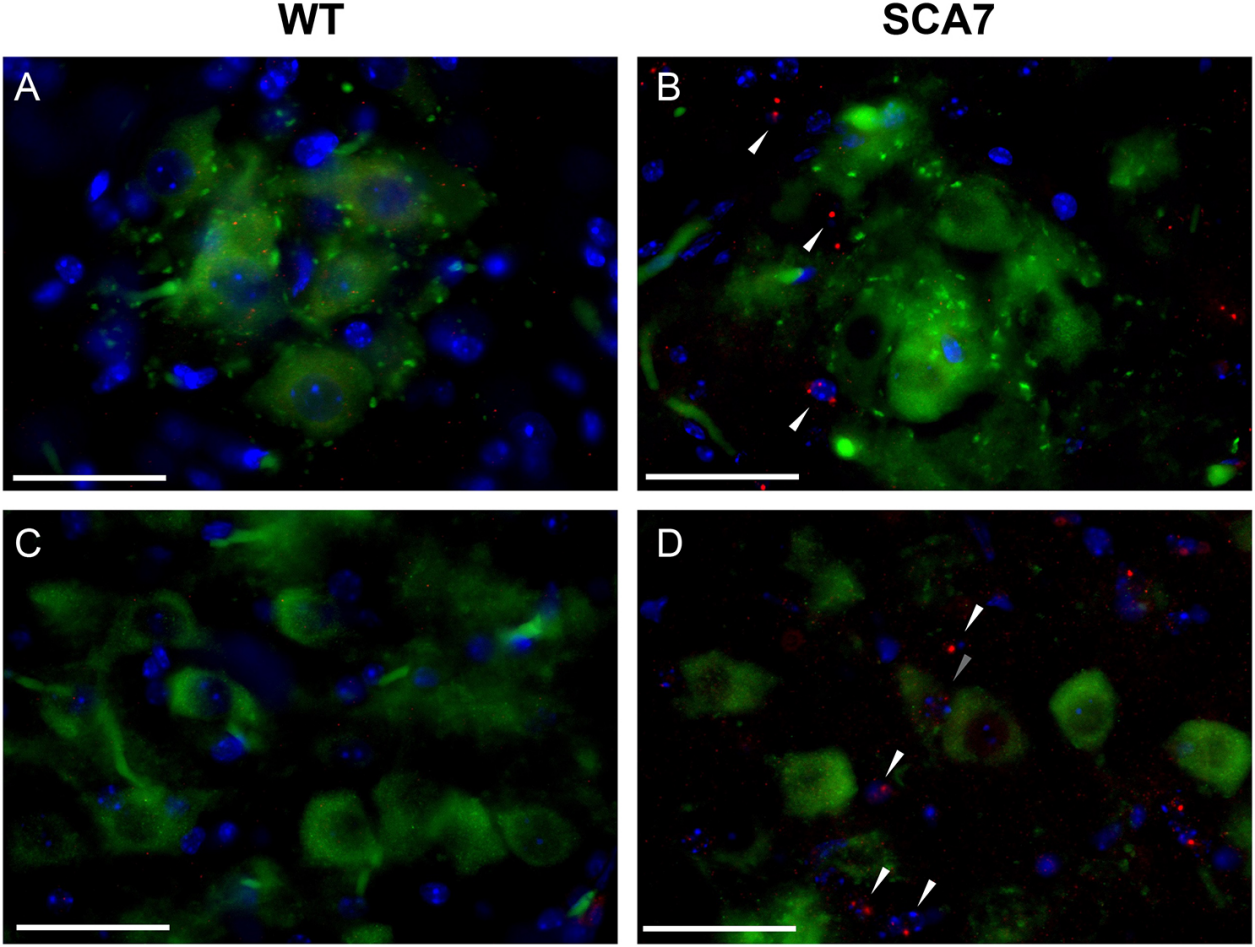
**SCA7 mice have an accumulation of ATXN7 protein in the nuclei of cells surrounding putative phrenic and hypoglossal motor neurons.** (A-D) Representative images of ChAT- (green), ATXN7- (red) and DAPI- (blue) stained cervical spinal cord and medulla of 10-week-old wild-type (WT) and SCA7 mice. Putative phrenic motor nucleus of wild-type (A) and (B) SCA7 mice. Hypoglossal motor nucleus of wild-type (C) and SCA7 (D) mice. ATXN7 aggregates are indicated with triangles. Scale bars: 40 µm.

The formation of nuclear inclusions of ataxin-7 protein (ATXN7) in neurons throughout the central nervous system, not limited to regions of severe neuronal loss, is a hallmark of SCA7 (Ansorge et al., 2004; Chort et al., 2013; Garden and La Spada, 2008; Holmberg, 1998; Yoo et al., 2003). To determine whether ataxin-7 also accumulates in phrenic and hypoglossal motor neurons and/or surrounding cells of SCA7 mice, cervical and medulla cross-sections were dual stained with anti-ATXN7 and anti-ChAT. Aggregates of ataxin-7 were observed within DAPI-labeled nuclei of cells near putative phrenic and hypoglossal motor neurons of SCA7 mice (Fig. 5B,D), but were absent in wild-type controls (Fig. 5A,C). In summary, motor neurons in key respiratory centers of SCA7-266Q mice reveal extensive pathology involving neurodegeneration, neuroinflammation and local accumulation of ataxin-7 protein.

### SCA7-266Q mice display ataxia by 8 weeks of age and fail to thrive

**Fig. 6.**
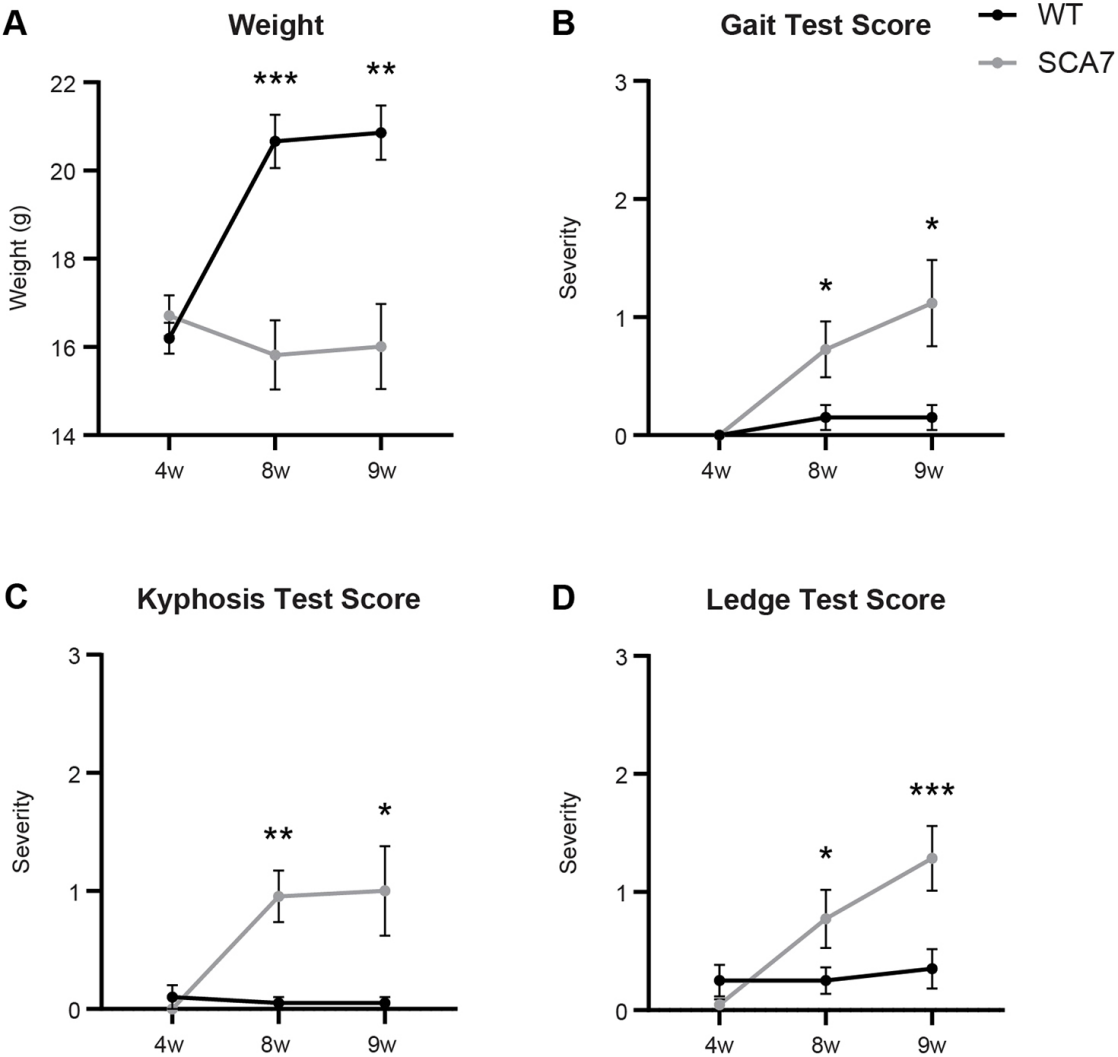
**SCA7 mice have reduced weights and increased motor and behavioral disorder at 8 and 9 weeks of age.** (A) Weights of wild-type (WT) and SCA7 mice at indicated ages. (B-D) Scores for gait (B), kyphosis (C) and ledge tests (D) at indicated ages. Data are mean±s.e.m. Statistical significance was determined using a mixed-model two-way ANOVA, followed by a Bonferroni's post-hoc analysis (*P<0.05; **P<0.01; ***P<0.001; 4 and 8 weeks, *n*=10 wild type, *n*=11 SCA7; 9 weeks, *n*=10 wild type, *n*=7 SCA7).

As previous studies have reported, at 4 weeks of age, before disease onset, there was no significant difference in weights between SCA7 and wild-type mice (Yoo et al., 2003). However, by 8 weeks, SCA7-266Q mice failed to thrive and had significantly lower weights than their age-matched wild-type littermates at 8 and 9 weeks of age (Fig. 6A; *P*=0.0001, *P*=0.0011). SCA7-266Q mice died between 8-11 weeks of age; their exact cause of death was unclear, but they displayed progressive weakness and we believe that respiratory insufficiency was a factor.

Neurobehavioral assessments, including ledge, gait, kyphosis and clasping tests, were used to track the onset and worsening of symptoms in SCA7-266Q mice. Although ledge and gait tests assess global sensorimotor function and are not specific to areas affected by motor neuron disease, they effectively encapsulate the progression of ataxia in similar mouse models (Becker et al., 2017; Niu et al., 2018; Pryor et al., 2014). Higher scores on the ledge and gait tests indicate broad deficits in motor coordination and/or function, whereas kyphosis, dorsal curvature of the spine, is a sign of muscle weakness (Guyenet et al., 2010). SCA7 and wild-type mice performed similarly at 4 weeks of age, earning average scores near zero (indicating no deficits) in all four tests (Fig. 6B-D; *P*>0.34 for all tests). Beginning at 8 weeks, SCA7 mice earned significantly higher scores for gait (Fig. 6B; *P*=0.0378), kyphosis (Fig. 6C; *P*=0.0058) and ledge tests (Fig. 6D; *P*=0.0310) than wild-type littermates, indicating worsening disease. We did not observe a statistically significant difference in clasping behavior between SCA7 and wild-type mice at any time point (results not shown).

Together, the weight and behavioral tests reveal that SCA7-266Q mice display a motor phenotype comparable to infantile-onset SCA7 patients, including progressive loss of motor coordination and function. In addition, SCA7-266Q mice exhibit a rapid onset of disease consistent with early-onset SCA7: although largely asymptomatic at 4 weeks, they show severe symptoms by 8 weeks, as previous studies of this mouse model have also reported (Niu et al., 2018; Noma et al., 2012; Stoyas et al., 2020; Yoo et al., 2003).

## DISCUSSION

Here, we show that SCA7-266Q knock-in mice have pronounced respiratory deficits that correlate with severe pathology in phrenic and hypoglossal motor nuclei. These respiratory deficits are progressive and result in an impaired ability to respond to hypercapnia and hypoxia at end-stage disease. Furthermore, there is neurodegeneration and neuroinflammation in the respiratory motor pools of SCA7-266Q mice. Collectively, these findings demonstrate that respiratory motor neuronal pathology likely contributes to death in SCA7-266Q mice, and may partially explain the respiratory pathology observed in human infantile-onset SCA7 (Niu et al., 2018; Noma et al., 2012; Stoyas et al., 2020; Ward et al., 2019; Yoo et al., 2003).

By 8 weeks, SCA7-266Q mice exhibit a dramatic decline in breathing function, as shown by significant reductions in measurements of respiratory output. Near end stage of disease, SCA7 mice can still maintain a minute ventilation comparable to wild-type littermates during eupneic breathing. This minute ventilation is achieved via a compensatory elevation in baseline breath frequency that suppresses respiratory insufficiency and is observed in mouse models of amyotrophic lateral sclerosis and other SCAs (Fusco et al., 2020; Orengo et al., 2018). However, when respiratory demand increases, SCA7 mice can no longer compensate to sustain adequate ventilation and their respiratory dysfunction becomes apparent.

In addition, SCA7-266Q mice nearing end stage show an increase in interbreath variability at baseline and spend a greater percentage of time in apnea during a respiratory challenge, both of which are suggestive of pathology in respiratory rhythm generators, such as the pre-Bötzinger complex (Rekling and Feldman, 1998). Interestingly, these findings deviate from earlier observations of other SCA mouse models, which revealed that regulatory breathing centers are spared in a mouse model of SCA1 (Orengo et al., 2018). Whether this difference can be attributed to disparities inherent in SCA1 and SCA7 pathology or to differences in the number of repeats, and thus disease severity, of the models is unclear and will require further investigation. Although respiratory failure requiring mechanical ventilation has been described in patients with SCA7 (Benton et al., 1998), central sleep apnea has not specifically been reported. However, central sleep apnea has been reported in other SCA patients, such as in patients with SCA6 (Rueda et al., 2016).

Immunohistochemical approaches reveal significant pathology in the putative phrenic and hypoglossal motor nuclei of SCA7-266Q mice. Clinically, phrenic and hypoglossal neurodegeneration may contribute to respiratory insufficiency, as well as dysphagia and aspiration pneumonia, respectively, in patients with SCA7 (Gousse et al., 2018). Degeneration of hypoglossal and spinal cord motor neurons was previously reported in human SCA7 patients (Ansorge et al., 2004; Seidel et al., 2012). Congruently, we find that significant loss of putative phrenic and hypoglossal motor neurons is a feature of SCA7 mice, and may play a role in their striking respiratory deficits.

The role of microglia in neurodegenerative diseases has been broadly described, and previous literature suggests that the pathogenesis of SCA7 may involve non-cell-autonomous pathways, with glial cells bringing about pathology in neighboring neurons responsible for motor control (Custer et al., 2006; Furrer et al., 2011; Garden et al., 2002; Lobsiger and Cleveland, 2007; Noma et al., 2012). However, these observations have primarily focused on the cerebellum, in which symptoms of ataxia originate. Because the most life-threatening symptoms of infantile SCA7 are likely respiratory in nature, we seek to widen the scope of attention to include microglia activity in respiratory areas. In SCA7-266Q mice, we find increased numbers of microglia that appear to have an activated morphology in the putative phrenic and hypoglossal motor pools.

ATXN7 accumulation is also observed qualitatively in cells surrounding the putative respiratory motor neurons. ATXN7 accumulation was previously described in spinal cord motor neurons and hypoglossal motor neurons of patients with SCA7 (Ansorge et al., 2004; Rüb et al., 2006), but has not been investigated fully in supporting cells of respiratory control centers. The pathogenesis of ATXN7 accumulation is still not clear and previous literature has noted that not all degenerated nuclei have intranuclear inclusions (Yoo et al., 2003), which may indicate that non-cell-autonomous pathways play a role in the pathology of SCA7 within the phrenic and hypoglossal motor nuclei. Because glial cells support proper motor neuron health, aggregation of mutant ATXN7 in these cells may contribute to motor neuron death and SCA7 pathogenesis. Similar phenomena are reported in amyotrophic lateral sclerosis and other neurodegenerative disorders, in which mutant synthesis of the affected protein in non-neuronal supporting cells accelerates disease progression (Beers et al., 2006; Boillee et al., 2006; Ilieva et al., 2009; Yamanaka et al., 2008a,b). We did not observe significant aggregation of ATXN7 within putative phrenic and hypoglossal motor neurons. This lack of neuronal accumulation is consistent with findings by Yoo et al. (2003) who originally described this model and found that nuclear inclusions appeared in the glial cells and not in the neurons of the spinal cord. Further studies will be required to determine the extent of ATXN7 accumulation in the cells surrounding the putative phrenic and hypoglossal motor neurons, and how these cells contribute to respiratory pathology.

In conclusion, SCA7-266Q mice have severe respiratory deficits and significant pathology in the putative phrenic and hypoglossal motor nuclei. Although upper airway dysfunction, respiratory insufficiency and respiratory failure have been reported in patients with SCA7, to our knowledge this is the first report that describes respiratory deficits and examines underlying respiratory motor neuron pathology in a mouse model of SCA7. Future studies should further elucidate the mechanisms of respiratory dysfunction in SCA7-266Q mice, and whether sex differences, impairments in chemosensitivity and respiratory muscle weakness or disruption of the neuromuscular junction contribute to the respiratory phenotype of SCA7. As upper airway dysfunction and respiratory insufficiency significantly contribute to morbidity and mortality in SCA7, our findings underscore the importance of assessing the impact of future therapies on respiratory function in this SCA7 mouse model.

## MATERIALS AND METHODS

### Animals

All procedures were approved by the Duke University Institutional Animal Care and Use Committee. SCA7-266Q knock-in mice (*Mus musculus*) were generated by the Huda Zoghbi Lab at Baylor College of Medicine, Houston, USA (Yoo et al., 2003), and bred by the laboratory of Dr Albert La Spada at Duke University. SCA7 mice were compared to *Atxn7^5Q/5Q^*, wild-type littermates. Animals were housed at the Duke University Division of Laboratory Animal Resources on a 12-h light/dark cycle with *ad libitum* access to food and water. Beginning at 7 weeks of age, mice were provided wet Purina Rodent Laboratory Chow (Purina, St Louis, MO, USA) and HydroGel (Clear H_2_O, Portland, ME, USA) packs to supplement their regular food and water supply. Mice were euthanized at a predetermined humane endpoint, when they could not right themselves after 15 s on their sides.

### Whole-body plethysmography

Breathing was assessed in unrestrained and unanesthetized mice via whole-body plethysmography (Buxco, DSI, St Paul, MN, USA) as described previously (Keeler et al., 2020; Stoica et al., 2017; Zieger et al., 2019). In brief, mice were placed into clear plexiglass chambers and exposed to normoxia (FiO_2_, 0.21; N_2_ balance) for 1.5 h. Following a stable 5-min baseline, mice were exposed to a 10-min hypercapnic/hypoxic challenge (FiCO_2_, 0.07; FiO_2_, 0.10; N_2_ balance). FinePointe software was used to analyze minute ventilation (MV), tidal volume (TV), breath frequency (f), peak inspiratory flow (PIF), peak expiratory flow (PEF), inspiratory time (Ti) and inspiratory flow rate (TV/Ti). To control for the effects of variation in body size, measurements of MV, TV and TV/Ti were divided by the weight of each mouse in grams. The quantity (NApnea), duration (TApnea) and percentage of time spent in apnea (PApnea) was also analyzed. Breaths were considered apneic if they were 1.5 s in duration or longer, or 200% longer than the previous breath. The coefficient of variation of the inspiratory time within the 5-min baseline was analyzed using GraphPad Prism. To track the progression of respiratory dysfunction, whole-body plethysmography was performed at 4 and 8 weeks, before and after disease onset, in ten wild-type (six males and four females) and ten SCA7 (six males and four females) mice.

### Behavioral tests

Behavioral tests were performed in wild-type and SCA7 mice at 4, 8 and 9 weeks of age. Neurobehavioral assessments were performed as described previously (Guyenet et al., 2010). This protocol grades the severity of ataxia in mice on a four-point scale (0 to 3, with 0 indicating an absence of pathology and 3 indicating the most severe pathology) in four distinct tests: a ledge test, a clasping test, a gait test and a test for kyphosis. In brief, for the ledge test, mice were placed on the ledge of their cage and their movement around the perimeter was analyzed. For the clasping test, mice were lifted by their tail and held for 10 s while clasping of their forelimbs and hind limbs was analyzed. For the gait test, mice were placed on a benchtop and their movements were analyzed. For the kyphosis test, the spinal curvature of each mouse while at rest and in motion was analyzed. Scoring was performed by a researcher blinded to genotypes, referencing specific scoring criteria for each test as described previously (Guyenet et al., 2010). Each test was performed in duplicate and the average score was recorded. At 4 and 8 weeks, ten wild-type (six males and four females) and 11 SCA7 (six males and five females) mice were tested. At 9 weeks, ten wild-type (six males and four females) and seven SCA7 (five males and two females) mice were tested, because several SCA7 mice died before 9 weeks of age.

### Tissue processing

Wild-type (*n*=10) and SCA7 (*n*=10) mice were anesthetized with inhaled isoflurane then euthanized via double thoracotomy followed by removal of the heart. Spinal cords and brainstems were harvested along with vertebrae and lower cranium, and postfixed for 48-72 h in 4% paraformaldehyde (PFA). The spinal cord and brainstem were extracted from the surrounding soft tissue and bone, and placed in 4% PFA for 24 h. The tissues were then transferred to 30% sucrose for dehydration and cryoprotection. Cervical spinal cords and medullas were embedded in Tissue Tek optimal cutting temperature compound (Sakura Finetek, Torrance, CA, 4583) and cut in cross-sections (40 μm) using a Leica CM3050 S cryostat. Sections were stored in 2% PFA.

### Immunohistochemistry

Every fourth section of the cervical spinal cord in the region of the putative phrenic motor nucleus and every third section of the medulla in the region of the hypoglossal motor nucleus was stained for choline acetyltransferase (ChAT) to label motor neurons. Putative phrenic motor neuron counts were assessed in six wild-type (three males and three females) and four SCA7 (two males and two females) mice (average age, 10 weeks±1 week). Hypoglossal motor neuron counts were assessed in four wild-type (three males and one female) and five SCA7 (three males and two females) mice (average age: 10 weeks±1 week). Free-floating sections were washed in PBS, quenched for 1 h, blocked with 10% normal horse serum for 1 h (Vector Laboratories, Burlingame, CA, USA, S-2000) and incubated overnight in primary antibody solution (1:100, polyclonal goat anti-ChAT, Millipore, Burlington, MA, USA, AB144P). Tissues were then washed and incubated in secondary antibody [1:200, biotin-SP-conjugated AffiniPure Donkey Anti-Goat IgG (H+L), Jackson ImmunoResearch, West Grove, PA, USA, 705-065-003] for 2 h. A Vectastain Avidin-Biotin Complex (ABC) Kit was applied for 2 h (Vector Laboratories, Burlingame, CA, USA, PK-4000) followed by 10 min with a 3,3′-Diaminobenzidine (DAB) Substrate Kit (Vector Laboratories, Burlingame, CA, USA, SK-415). Sections were then mounted onto charged slides, allowed to dry overnight and counterstained with 0.1% Cresyl Violet acetate.

For microglia quantification, cross-sections from the regions containing the phrenic and hypoglossal motor pools in four wild-type (two males and two females) and four SCA7 (two males and two females) mice were dual-stained with anti-ChAT (1:250, Millipore, Burlington, MA, USA, AB144P-1ML) and anti-Iba1 (1:300, FUJIFILM Wako Pure Chemical Corporation, Tokyo 019-19741). On day 1, sections were processed as described above. On day 2, sections were washed then incubated for 2 h in a secondary antibody solution consisting of 1:500 anti-goat IgG Alexa Fluor 488 (Invitrogen, Carlsbad, CA, USA, A32814) and 1:500 anti-rabbit IgG Alexa Fluor 594 (Invitrogen, Carlsbad, CA, USA, A32754). Sections were mounted on Vectashield Antifade Mounting Medium with DAPI (Vector Laboratories, Burlingame, CA, USA). Cervical and medulla cross-sections in the regions of the putative phrenic and hypoglossal motor nuclei were dual stained with anti-ChAT and anti-ataxin-7 polyclonal antibody (1:500, Invitrogen, Carlsbad, CA, USA, PA1-749) using the same protocol. For negative control staining, tissues were stained with secondary antibodies only.

### Imaging and cell counting

Images of cervical spinal cord and medulla sections were acquired using an ECHO Revolve microscope. Putative phrenic and hypoglossal motor pools and motor neurons were identified in each section based on size, morphology and location, and quantified as described previously (Boulenguez et al., 2007; Goshgarian and Rafols, 1981; Nichols et al., 2015). ChAT^+^ cells with the morphology of motor neurons, and Iba1^+^ cells in the regions of the putative phrenic and hypoglossal motor pools, were counted by two researchers blinded to genotypes.

### Statistics

Statistical analyses were performed using GraphPad Prism. Statistical significance was set *a priori* at *P*≤0.05. Behavioral tests and weights were analyzed using a mixed-model two-way ANOVA. Plethysmography and apnea data were analyzed using a two-way repeated measures (RM) ANOVA. Bonferroni's multiple comparisons test was used for post-hoc analysis. The coefficient of variation of inspiratory time, motor neuron counts and microglia counts were each analyzed using an unpaired Student's *t*-test. All data are presented as mean±s.e.m.
